# Dapagliflozin improves treatment satisfaction in overweight patients with type 2 diabetes mellitus: a patient reported outcome study (PRO study)

**DOI:** 10.1186/s13098-018-0313-x

**Published:** 2018-03-01

**Authors:** Hiroki Nakajima, Sadanori Okada, Takako Mohri, Eiichiro Kanda, Naoyuki Inaba, Yoko Hirasawa, Hiroaki Seino, Hisamoto Kuroda, Toru Hiyoshi, Tetsuji Niiya, Hitoshi Ishii

**Affiliations:** 1 Department of Diabetology, Nara Medical University, 840 Shijo-cho, Kashihara, Nara 634-8522 Japan; 2 Department of Nephrology, Tokyo Kyosai Hospital, Tokyo, Japan; 3 Life Science and Bioethics Research Center, Tokyo Medical and Dental University, Tokyo, Japan; 4 Department of Metabolism & Endocrinology, Shizuoka Saiseikai General Hospital, Shizuoka, Japan; 5 Plumeria DM Clinic, Shizuoka, Japan; 6 Seino Internal Medicine Clinic, Koriyama, Japan; 7 Green Clinic, Simotsuga, Japan; 8 Japanese Red Cross Medical Center, Tokyo, Japan; 9 Department of Internal Medicine, Matsuyama Shimin Hospital, Matsuyama, Japan

**Keywords:** Type 2 diabetes mellitus, Oral hypoglycemic agent, Sodium glucose cotransporters 2 inhibitors, SGLT2 inhibitors, Dapagliflozin, Treatment satisfaction, Oral Hypoglycemic Agent-Questionnaire, Body weight, Patient reported outcome, Quality of life

## Abstract

**Background:**

The benefits of sodium glucose cotransporters 2 (SGLT2) inhibitors in patients with type 2 diabetes mellitus include plasma glucose control, reduction in body weight and blood pressure, and low risk of hypoglycemia, although they may also cause genitourinary infections, polyuria, or volume depletion. It is not clear whether dapagliflozin, an SGLT2 inhibitor, improves treatment satisfaction among patients in a comprehensive way despite the negative side effects. This study assessed the effect of dapagliflozin on glycosylated hemoglobin (HbA1c), body weight, and treatment satisfaction in overweight patients with type 2 diabetes mellitus treated with oral hypoglycemic agents.

**Methods:**

This multicenter, open-label, single-arm observational study included patients with type 2 diabetes mellitus administering dapagliflozin 5 or 10 mg per day for 14 weeks. Changes in treatment satisfaction were evaluated using a new version of the Oral Hypoglycemic Agent-Questionnaire (OHA-Q ver. 2) consisting of 23 items. Correlation between treatment satisfaction and HbA1c levels and body weight were analyzed using the Spearman’s rank-correlation coefficient.

**Results:**

Of the 221 patients enrolled, 188 completed the study. Mean HbA1c decreased from 7.8 ± 0.7% (62.1 ± 7.5 mmol/mol) to 7.3 ± 0.8% (55.9 ± 8.7 mmol/mol) (change − 0.6 ± 0.7%, *P* < 0.001) and body weight decreased from 82.5 ± 14.6 to 80.7 ± 14.8 kg (change − 2.3 ± 2.8 kg, *P* < 0.001). OHA-Q ver. 2 was validated as well, the mean OHA-Q ver. 2 total score increased from 44.3 ± 9.4 to 46.6 ± 9.8 (best score 69, worst score 0; change 2.3 ± 6.6, *P* < 0.001). The change in body weight significantly correlated with the OHA-Q ver. 2 total score (Spearman’s *ρ* = − 0.17, *P* = 0.035). The change in HbA1c levels significantly correlated with the satisfaction subscale score (Spearman’s *ρ* = − 0.19, *P* = 0.011).

**Conclusions:**

Dapagliflozin significantly improved treatment satisfaction among patients with type 2 diabetes mellitus for 14 weeks. Body weight loss significantly correlated with treatment satisfaction.

*Trial registration* UMIN-CTR: UMIN000016304

**Electronic supplementary material:**

The online version of this article (10.1186/s13098-018-0313-x) contains supplementary material, which is available to authorized users.

## Background

Diabetes mellitus increases the risk of microvascular and macrovascular events [1, 2]. Abnormal glycemic metabolism, such as hyperglycemia or possibly, large daily glucose fluctuations, is a major risk factor for these complications [3]. Therefore, control of plasma glucose is a primary objective in the daily treatment of diabetes mellitus. A range of oral hypoglycemic agents (OHAs) are available, such as biguanides, thiazolidinediones, sulphonylureas, glinides, α-glucosidase inhibitors, and dipeptidyl peptidase-4 (DPP-4) inhibitors. Recently, sodium-glucose co-transporter 2 (SGLT2) inhibitors have become available.

The mechanism of action of SGLT2 inhibitors is independent of insulin action, these drugs have a low risk of hypoglycemia, and reduce body weight, blood pressure, and serum triglyceride level [4, 5]. Large scale trials (EMPA-REG OUTCOME, CANVAS, and CANVAS-R) established the safety of SGLT2 inhibitors and have demonstrated a reduction in the frequency of cardiovascular events and risk of renal failure in patients with type 2 diabetes mellitus (T2DM), with an elevated risk of cardiovascular disease and an increased risk of amputation [6–8]. A further advantage of SGLT2 inhibitors is that they can be combined with any other OHAs, due to their different mechanism of action. Therefore, SGLT2 inhibitors could benefit patients with inadequate plasma glucose control with conventional therapies. However, adverse events, such as increased polyuria/pollakiuria, thirst, urinary tract infection, and genital infection have been commonly reported with SGLT2 inhibitor use [9–16]. Use of SGLT2 inhibitors such as dapagliflozin has the potential to positively impact treatment satisfaction.

Treatment satisfaction and health-related quality of life (HRQOL) are important for successful treatment of diabetes [17]. It has been reported that improved quality of life (QOL) results in improved adherence with medication [18] and poor adherence contributes to poor glycemic control [19]. Treatment of diabetes is long term and patients need to manage therapeutic regimes independently. Treatment can impair the QOL of patients. Some OHA cause hypoglycemia and body weight gain, which potentially impair motivation for treatment or patient’s QOL [20]. It has been reported that not only clinical assessment but also patient reported outcome (PRO) are important in the evaluation of treatment outcome [21]. A PRO is a health outcome directly reported by the patients experiencing it. The value of PROs has been increasingly recognized over recent years and the US Food and Drug Administration has released a definitive guidance on the use of PRO [22].

Recently, PROs were measured among patients with T2DM treated with SGLT2 inhibitors using several questionnaires for measuring patients’ QOL and treatment satisfaction [23–28]. However, there are no studies investigating the effect of SGLT2 inhibitors on treatment satisfaction, including the effect of medication side effects and there is no OHA-specific satisfaction questionnaire. The Oral Hypoglycemic Agent-Questionnaire (OHA-Q) was designed specifically for patients treated with oral hypoglycemic agents [29]. It consists of 3 subscales, “treatment convenience”, “somatic symptom” and “satisfaction”. The OHA-Q is the only satisfaction instrument that specific to oral hypoglycemic treatment [17]. It can evaluate treatment satisfaction, including unique side effects to OHA. SGLT2 inhibitors became available after the development of the OHA-Q. Therefore, it is necessary to insert questions in the OHA-Q regarding frequent side effects of SGLT2 inhibitors.

The objective of this study was to evaluate the changes in treatment satisfaction of overweight patients with T2DM who added on dapagliflozin among 14 weeks. Because SGLT2 inhibitors had an effect of body weight loss, we selected overweight patients with T2DM as subjects. OHA-Q ver. 2 was used to address both advantage and disadvantage (common side effect) of SGLT2 inhibitors.

## Methods

### Study design

A 14-week, multicenter, open-label, single-arm observational study was conducted between January 2015 and May 2017 at 29 sites across Japan listed in Additional file 1). Enrolled patients received dapagliflozin 5 mg once daily; if glycemic control was inadequate, the dose was increased to 10 mg once daily. We did not place any limitation on the time of use and the dosage of dapagliflozin because this was an observational study; the timing and dosage were entrusted to the attending physicians. We collected treatment satisfaction scores using the OHA-Q ver. 2 (see “Development of OHA-Q ver. 2” section) and the following clinical and biochemical parameters: body weight, abdominal circumference, body composition, blood pressure, fasting blood glucose levels, glycosylated hemoglobin (HbA1c) levels, hepatic enzyme levels, renal function, lipid profile, hematological values, and urinary findings (urinary albumin and creatinine) at baseline and 14 weeks after the administration of dapagliflozin was commenced. The waist measurement was made by holding the tape measure at the level of the umbilicus at the end of a normal expiration, and bringing it around the waist in the upright position.

The study protocol was registered with the University Hospital Medical Information Network Clinical Trials Registry (UMIN-CTR: UMIN000016304) prior to the commencement of the study. We adhered to the “Ethical Guidelines for Medical and Health Research Involving Human Subjects” issued by the Japanese government after receiving approval from the ethical committees at each of the participating medical facilities. This study was conducted in accordance with the ethical standards laid down in the Declaration of Helsinki and its later amendments. Written informed consent was obtained from all patients after an explanation of the study. All personal information was anonymized.

### Patients

The inclusion criteria were as follows: a diagnosis of T2DM; HbA1c 6.5% ≤ and < 10.0%, at least 12 weeks of treatment with antidiabetic drugs, other than SGLT2 inhibitors, in addition to diet and exercise, prior to the commencement of the study; patients started dapagliflozin medication in addition to other antidiabetic drugs; no prior use of insulin or glucagon-like peptide-1 (GLP-1) receptor agonists; body mass index (BMI) > 25 kg/m^2^; estimated glomerular filtration rate > 45 ml/min; and age between 20 and 70 years. Exclusion criteria were: severe hypoglycemia, unstable blood pressure or lipid abnormalities within 12 months of signing the consent form; history of myocardial infarction, angina or cerebral infarction; patients with New York Heart Association class III or above; serum creatinine > 1.4 mg/dl in male and > 1.2 mg/dl in female participants; aspartate transaminase (AST) ≤ 100 IU/l; dementia.

### Development of OHA-Q ver. 2

The original OHA-Q consisted of 20 items and three subscales “treatment convenience”, “somatic symptom” and “satisfaction” [29]. We developed the revised OHA-Q (OHA-Q ver. 2), including items regarding known SGLT2 inhibitor side-effects. We added 3 new items relevant to SGLT2 inhibitors: frequent urination, thirst, and discomfort with urination or genital pruritus. The items identified for the development of the OHA-Q ver. 2 are presented in Additional file 2. Scores in the OHA-Q ver. 2 were calculated according to the original OHA-Q scores as follows: answers for each question were converted to values between 0 and 3 (answer numbers 1, 2, 3, and 4 were converted to scores 3, 2, 1, and 0, respectively, with higher scores indicating a higher satisfaction). The subscale structure of the OHA-Q ver. 2 was defined after examining the results of factor analysis and Cronbach’s α coefficient.

### Data collection

The questionnaire of the OHA-Q ver. 2 was completed by participants in private, to avoid any influence by physicians and medical care providers. The indices of body composition were measured using Tanita DC320 (Tokyo, Japan). The biochemical markers were measured using fasting blood samples and urine samples collected in hospital at baseline and at week 14.

### Study endpoints

The primary endpoints were changes in OHA-Q ver. 2 scores and correlations between changes in HbA1c levels and OHA-Q ver. 2 scores and between changes in body weight and the OHA-Q ver. 2 scores. The secondary endpoints were frequency of adverse events observed throughout the study and changes in body weight, body composition, HbA1c, and lipid metabolism.

### Sample size

Regarding the first primary endpoint, changes in OHA-Q ver. 2 scores, reports on the original OHA-Q were used as a reference for sample size calculation, due to the lack of reports on treatment satisfaction scores in OHA-Q ver. 2. Mean item scores of the original OHA-Q in Japanese patients with T2DM were reported as 2.22 ± 0.78 (mean ± standard deviation [SD]) [29]. Accordingly, we assumed a baseline score of 2.22 ± 0.78 and a 10% improvement over 14 weeks from the baseline value, resulting in a score of 2.44 at week 14 in this study. Furthermore, correlations between the scores before and after treatment were assumed as 0.1. Based on these assumptions, the number of cases required to detect a significant difference in OHA-Q scores between before and after the dapagliflozin therapy under the conditions of two-sided *P* value of 5% and with a power of 80% was 185 patients. Assuming a 10% dropout rate, the number of enrolled patients required was 206. For the second primary endpoint of correlations between changes in HbA1c/body weight and OHA-Q ver. 2 scores, assuming that a correlation coefficient 0.2 would be detected, 194 patients were required under the conditions of two-sided *P* value of 5% and with a power of 80%. Assuming a 10% dropout rate, the number of enrolled patients required was 216. Finally, a target number of 220 patients was set for this study.

### Statistical analysis

All analyses, except for safety analysis, were performed on the full analysis set (FAS), which excluded patients with missing data or questionnaire information at baseline or week 14 and those administered further drugs in the course of the study.

In order to develop the OHA-Q ver. 2, we analyzed the structure of the questionnaire with 23 items (20 items in the original OHA-Q + new 3 items) using factor analysis, in which we applied the principal factor method with promax rotation. The number of factors was set at 3, as with the original OHA-Q. Internal consistency of the overall items and the items in each subscale was assessed by Cronbach’s α coefficient.

Changes at 14 weeks from baseline were tested using the one-sample *t* test or the Wilcoxon signed-rank test, according to data distribution, including OHA-Q ver. 2 scores and clinical and biochemical parameters. Correlation analyses were performed using the Spearman’s rank correlation coefficient. All statistical tests were two-sided with a 5% significance level. All analyses were performed using the SAS version 9.3 (SAS Institute, Cary, NC).

## Results

### Study population and patient characteristics

**Fig. 1:**
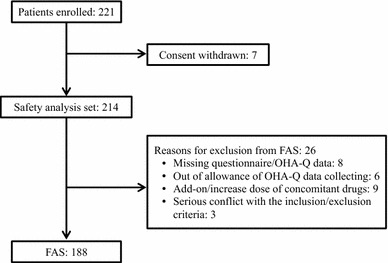
Flowchart (FAS, full analysis set; OHA-Q, oral hypoglycemic agent questionnaire)

The study flow is shown in Fig. 1. A total of 221 patients were enrolled in this study. Seven patients were excluded due to withdrawal of consent; therefore, 214 patients were included in the safety analysis set. Of these, 26 patients were excluded from the FAS for the following reasons: missing values in the OHA-Q ver. 2, measurements taken outside the pre-specified time allowance of week 14, increase in drug dosage or introduction of further antidiabetic drugs to the treatment regime, use of insulin, and treatment without hypoglycemic agents at the time of enrollment. Finally, the FAS included data from 188 patients.

Table 1 shows the patients characteristics at the baseline. The study included 123 male (65.4%) and 65 female (34.6%) participants. The following parameters, presented as mean ± SD, were measured: age, 51.1 ± 9.4 years; HbA1c, 7.8 ± 0.7%; body weight, 82.5 ± 14.6 kg; BMI, 30.0 ± 4.4 kg/m^2^; and duration of diabetes, 7.3 ± 4.9 years. At baseline, 185 (98.4%) patients were treated with at least one OHA, with biguanides being used most frequently (72.9%), followed by DPP-4 inhibitors (68.6%), sulfonylureas (36.2%), thiazolidinediones (16.5%), α-glucosidase inhibitors (14.4%), and glinides (3.7%). Dapagliflozin was administered at a dose of 5 mg/day in the majority of patients (98.4%) at baseline, and the dose was not changed during the study, except for 4 patients in whom the dose was increased from 5 to 10 mg/day. The mean dapagliflozin dose was 5.1 ± 0.6 mg/day at baseline and 5.2 ± 0.9 mg/day at week 14.

**Table 1:** Baseline patient characteristics

Variable	Values
Age (years)	51.1 ± 9.4
Sex	
Male	123 (65.4)
Female	65 (34.6)
Duration of diabetes (years)	7.3 ± 4.9
Body weight (kg)	82.5 ± 14.6
BMI (kg/m^2^)	30.0 ± 4.4
Systolic blood pressure (mmHg)	132.3 ± 14.1
Diastolic blood pressure (mmHg)	80.6 ± 11.9
HbA1c (NGSP, %)	7.8 ± 0.7
HbA1c (mmol/mol)	62.1 ± 7.5
Fasting plasma glucose (mg/dl)	156.2 ± 39.6
Current smokers	50 (26.7)
Drinking	89 (47.3)
eGFR (ml/min/1.73 m^2^)	
90 ≤	65 (37.6)
60 ≤, < 90	92 (53.2)
45 ≤, < 60	15 (8.7)
< 45	1 (0.6)
Complications	
Diabetic retinopathy	22 (11.8)
Simple	13 (7.0)
Pre-proliferative	3 (1.6)
Proliferative	6 (3.2)
Diabetic nephropathy	53 (28.2)
Diabetic neuropathy	20 (10.6)
Macrovascular complications	0 (0.0)
Kidney disease	5 (2.7)
Liver disease	51 (27.1)
Hypertension	108 (57.4)
Dyslipidemia	125 (66.5)
Oral hypoglycemic agents	185 (98.4)
Sulphonylureas	68 (36.2)
Biguanides	137 (72.9)
α-Glucosidase inhibitors	27 (14.4)
Glinides	7 (3.7)
DPP-4 inhibitors	129 (68.6)
Thiazolidinediones	31 (16.5)
Antihypertensive agents	102 (54.3)
Antidyslipidemic agents	96 (51.1)
Antithrombotic agents	6 (3.2)

Data are shown as mean ± SD or as the number of patients (%)

*HbA1c* glycosylated hemoglobin, *DPP*-*4* dipeptidyl peptidase-4

### Validation of OHA-Q ver. 2

Table 2 presents the results of factor analysis for the OHA-Q ver. 2 with 23 items. Of the 23 items, 22 were divided into 3 factors, for which each factor loading was over 0.3. Candidate subscales and items are shown in Table 2 in italics. Based on the structure of the subscales in the original OHA-Q [29], we considered that factors 1, 2, and 3 correspond to the subscale 1 “treatment convenience”, subscale 2 “somatic symptom” and subscale 3 “satisfaction”, respectively. The 3 new items were included in the “somatic symptom” subscale. Although factor loading of item 2 for factor 1 was low (0.099), we included it into the “treatment convenience” subscale as with the original OHA-Q which was validated and reproduced in the previous study [29]. The “treatment convenience” subscale consisted of items 1–9, the “somatic symptom” subscale consisted of items 11–21, and the “satisfaction” subscale consisted of items 10, 22, and 23.

**Table 2:** Factor analysis

Items	Factor loading		
	Factor 1	Factor 2	Factor 3
1. Missed dose	*0.549*	− 0.166	0.149
2. Difficulty swallowing	*0.099*	0.272	0.161
3. Carrying and preparing for taking the agent	*0.589*	0.034	0.158
4. People around the patient	*0.347*	− 0.015	0.218
5. Following the meal schedule	*0.819*	0.058	− 0.145
6. Interval between taking the agent and a meal	*0.933*	− 0.034	− 0.140
7. Compliance with treatment schedule	*0.885*	0.008	− 0.088
8. Number of doses	*0.660*	0.048	0.119
9. Taking the agent at a place other than home	*0.507*	0.101	0.187
10. Desire to continue the treatment	0.213	− 0.044	*0.562*
11. Rumbling stomach	0.297	*0.302*	0.031
12. Diarrhea	0.142	*0.430*	0.014
13. Constipation	− 0.066	*0.634*	− 0.078
14. Increase in body weight	− 0.159	*0.374*	0.109
15. Tendency to become hungry easily	0.068	*0.497*	0.104
16. Nausea	0.110	*0.529*	− 0.040
17. Bodily swelling	0.041	*0.599*	− 0.103
18. Hypoglycemia	− 0.070	*0.382*	0.187
19. Frequent urination	0.117	*0.317*	0.172
20. Thirst	0.116	*0.435*	0.170
21. Discomfort with urination or genital pruritus	− 0.031	*0.503*	0.030
22. Glycemic control	− 0.133	0.105	*0.542*
23. Satisfaction with the current agent	0.128	0.035	*0.735*

The principal factor method with three-factor promax rotation was applied; *n* = 188; Values of factor loadings attributed subscale are shown in italics

Based on factor analysis results, Cronbach’s α coefficients were calculated for total score and the 3 candidate subscales (Table 3); the α coefficients ranged from 0.67 to 0.88, indicating good internal consistency of the total and candidate subscales. Therefore, we used the 23 items and the OHA-Q ver. 2 subscale structure in the following analyses.

**Table 3:** Cronbach’s α

	No. of items	Cronbach’s α coefficient
Subscale 1: Treatment convenience	9	0.87
Subscale 2: Somatic symptom	11	0.79
Subscale 3: Satisfaction	3	0.67
Total	23	0.88

*n* = 188

Total score and subscale scores were calculated as the sum of the scores after the conversion of each item score. The score ranges were as follows: each item 0–3, total score 0–69 (23 items), subscale 1 “treatment convenience” 0–27 (9 items), subscale 2 “somatic symptom” 0–33 (11 items), and subscale 3 “satisfaction” 0–9 (3 items).

### Effects of dapagliflozin on OHA-Q ver. 2 scores

Changes in OHA-Q ver. 2 scores at baseline and at week 14 are shown in Table 4. Significant increases were found in 11 items (item 1, 2, 8, 9, 12, 14, 16, 17, 18, 22, and 23), and a significant decrease was found only for item 19. The remaining 11 items did not show significant changes. The total score significantly increased from 44.31 ± 9.43 to 46.62 ± 9.83 (change: 2.31 ± 6.60, *P* < 0.001). Subscale 2 “somatic symptom” and 3 “satisfaction” significantly increased from 20.14 ± 5.28 to 21.36 ± 5.42 (change: 1.22 ± 4.41, *P* < 0.001), and from 5.09 ± 1.77 to 5.78 ± 1.67 (change: 0.70 ± 1.72, *P* < 0.001), respectively. Subscale 1 “treatment convenience” increase from 19.09 ± 4.78 to 19.48 ± 4.97 was not significant (change 0.39 ± 3.02, *P* = 0.08).

**Table 4:** Scores of OHA-Q ver. 2

Items	Baseline	Week 14	Change	P value
1. Missed dose	2.05 ± 0.77	2.21 ± 0.73	0.16 ± 0.65	< 0.001
2. Difficulty swallowing	2.62 ± 0.59	2.71 ± 0.54	0.09 ± 0.55	0.026
3. Carrying and preparing for taking the agent	2.29 ± 0.78	2.22 ± 0.83	− 0.07 ± 0.73	0.20
4. People around the patient	2.31 ± 0.85	2.31 ± 0.82	0.00 ± 0.60	1.00
5. Following the meal schedule	1.81 ± 0.92	1.81 ± 0.92	− 0.01 ± 0.79	0.93
6. Interval between taking the agent and a meal	1.77 ± 0.94	1.75 ± 0.90	− 0.02 ± 0.88	0.80
7. Compliance with treatment schedule	1.90 ± 0.92	1.87 ± 0.89	− 0.03 ± 0.80	0.59
8. Number of doses	2.23 ± 0.77	2.36 ± 0.71	0.13 ± 0.73	0.018
9. Taking the agent at a place other than home	2.10 ± 0.75	2.23 ± 0.73	0.13 ± 0.75	0.016
10. Desire to continue the treatment	1.77 ± 0.81	1.89 ± 0.84	0.12 ± 0.85	0.06
11. Rumbling stomach	1.95 ± 0.86	2.04 ± 0.87	0.09 ± 0.93	0.19
12. Diarrhea	2.08 ± 0.85	2.29 ± 0.85	0.21 ± 0.94	0.003
13. Constipation	2.05 ± 0.87	2.00 ± 0.99	− 0.05 ± 0.86	0.40
14. Increase in body weight	0.86 ± 0.91	1.48 ± 1.04	0.62 ± 1.01	< 0.001
15. Tendency to become hungry easily	1.73 ± 0.83	1.74 ± 0.82	0.02 ± 0.82	0.79
16. Nausea	1.89 ± 0.95	2.13 ± 0.85	0.24 ± 0.87	< 0.001
17. Bodily swelling	1.83 ± 0.93	2.13 ± 0.85	0.30 ± 0.82	< 0.001
18. Hypoglycemia	2.00 ± 0.78	2.11 ± 0.75	0.11 ± 0.74	0.040
19. Frequent urination	1.78 ± 0.84	1.57 ± 0.89	− 0.20 ± 0.94	0.004
20. Thirst	1.74 ± 0.79	1.66 ± 0.83	− 0.07 ± 0.86	0.24
21. Discomfort with urination or genital pruritus	2.22 ± 0.83	2.19 ± 0.82	− 0.03 ± 0.89	0.62
22. Glycemic control	1.43 ± 0.81	1.80 ± 0.71	0.37 ± 0.80	< 0.001
23. Satisfaction with the current agent	1.88 ± 0.68	2.10 ± 0.58	0.21 ± 0.70	< 0.001
Subscale 1: Treatment convenience	19.09 ± 4.78	19.48 ± 4.97	0.39 ± 3.02	0.08
Subscale 2: Somatic symptom	20.14 ± 5.28	21.36 ± 5.42	1.22 ± 4.41	< 0.001
Subscale 3: Satisfaction	5.09 ± 1.77	5.78 ± 1.67	0.70 ± 1.72	< 0.001
Total	44.31 ± 9.43	46.62 ± 9.83	2.31 ± 6.60	< 0.001

Data are shown as mean ± SD; *n* = 188; subscale 1: treatment convenience (score range 0–27); subscale 2: somatic symptom (score range 0–33); subscale 3: satisfaction (score range 0–9); total (score range 0–69)

*OHA*-*Q* Oral Hypoglycemic Agent-Questionnaire

### Correlations between changes in HbA1c or body weight and OHA-Q ver. 2 scores

Table 5 shows the results of correlation analysis between changes in HbA1c/body weight and OHA-Q ver. 2 scores. Significant correlations were detected between changes in HbA1c and subscale of “satisfaction” (Spearman’s *ρ* = − 0.19, *P* = 0.011), changes in body weight and subscale of “satisfaction” (Spearman’s *ρ* = − 0.22, *P* = 0.005), and changes in body weight and total score (Spearman’s *ρ* = − 0.17, *P* = 0.035). HbA1c did not correlate with the OHA-Q ver. 2 total score (Spearman’s *ρ* = 0.01, *P* = 0.89).

**Table 5:** Correlations between changes in HbA1c, body weight, and OHA-Q ver. 2 scores

Variable 1	Variable 2	*n*	Correlation coefficient	*P* value
HbA1c change	Subscale 1 score change	178	0.07	0.37
	Subscale 2 score change	178	0.05	0.54
	Subscale 3 score change	178	− 0.19	0.011
	Total score change	178	0.01	0.89
Body weight change	Subscale 1 score change	160	− 0.07	0.36
	Subscale 2 score change	160	− 0.12	0.14
	Subscale 3 score change	160	− 0.22	0.005
	Total score change	160	− 0.17	0.035

All of the variable 2 entries are scores of OHA-Q ver. 2; correlation coefficients and *P* values are the Spearman’s rank correlation coefficient; subscale 1: treatment convenience; subscale 2: somatic symptom; subscale 3: satisfaction

*HbA1c* glycosylated hemoglobin, *OHA*-*Q* Oral Hypoglycemic Agent-Questionnaire

### Effects of dapagliflozin on clinical and biochemical parameters

Changes in the clinical and biochemical parameters are shown in Table 6. HbA1c was significantly reduced (− 0.6 ± 0.7%, *P* < 0.001). Body weight, abdominal circumference, and body fat also showed significant reductions (− 2.3 ± 2.8 kg, − 1.7 ± 4.5 cm and − 1.0 ± 2.7 kg, respectively; all *P* < 0.001). A significant reduction was observed in AST levels (− 5.1 ± 12.9 IU/l, *P* < 0.001), alanine aminotransferase (ALT) levels (− 8.4 ± 19.2 IU/l, *P* < 0.001), uric acid levels (− 0.4 ± 0.8 mg/dl, *P* < 0.001), and systolic blood pressure (− 2.7 ± 13.7 mmHg, *P* = 0.011). Significant increases in the hematocrit were observed (2.4 ± 2.6%, *P* < 0.001).

**Table 6:** Clinical parameters

Parameters	Baseline	Week 14	Change	*P* value
HbA1c (NGSP, %)	7.8 ± 0.7 (184)	7.3 ± 0.8 (181)	− 0.6 ± 0.7 (178)	< 0.001
HbA1c (mmol/mol)	62.1 ± 7.5 (184)	55.9 ± 8.7 (181)	− 6.2 ± 7.5 (178)	< 0.001
Fasting plasma glucose (mg/dl)	156.2 ± 39.6 (136)	137.2 ± 29.9 (147)	− 18.5 ± 39.5 (126)	< 0.001
Body weight (kg)	82.5 ± 14.6 (175)	80.7 ± 14.8 (173)	− 2.3 ± 2.8 (160)	< 0.001
BMI (kg/m^2^)	30.0 ± 4.4 (175)	29.3 ± 4.4 (172)	− 0.8 ± 1.0 (160)	< 0.001
Abdominal circumference (cm)	100.0 ± 11.6 (184)	98.3 ± 11.1 (178)	− 1.7 ± 4.5 (175)	< 0.001
Body fat percentage (%)	33.5 ± 7.7 (172)	33.7 ± 7.9 (172)	− 0.4 ± 2.4 (156)	0.06
Body fat (kg)	27.8 ± 8.8 (172)	27.4 ± 9.1 (171)	− 1.0 ± 2.7 (156)	< 0.001
Lean body mass (kg)	54.8 ± 10.9 (172)	53.3 ± 10.8 (171)	− 1.3 ± 2.3 (156)	< 0.001
Muscle mass (kg)	51.8 ± 10.4 (172)	50.5 ± 10.3 (172)	− 1.2 ± 2.2 (156)	< 0.001
Body water (kg)	36.7 ± 6.3 (172)	35.8 ± 6.4 (172)	− 0.8 ± 1.9 (156)	< 0.001
Bone mass (kg)	3.0 ± 0.5 (172)	2.9 ± 0.5 (171)	− 0.1 ± 0.2 (156)	< 0.001
Basal metabolic rate (kcal)	1585.8 ± 296.2 (172)	1543.2 ± 292.4 (171)	− 41.5 ± 73.0 (156)	< 0.001
TC (mg/dl)	184.9 ± 33.1 (146)	185.9 ± 32.0 (158)	− 0.1 ± 24.9 (141)	0.94
HDL-C (mg/dl)	47.1 ± 11.1 (166)	47.9 ± 10.9 (169)	0.9 ± 6.3 (158)	0.08
LDL-C (mg/dl)	108.3 ± 29.8 (129)	108.5 ± 27.1 (141)	− 1.1 ± 22.0 (118)	0.59
TG (mg/dl)	135.0 [99.0, 178.0] (133)	127.0 [93.0, 174.0] (145)	0.0 [− 30.0, 21.0] (123)	0.35
AST (IU/l)	32.5 ± 18.4 (171)	27.4 ± 14.0 (167)	− 5.1 ± 12.9 (160)	< 0.001
ALT (IU/l)	46.0 ± 30.4 (171)	37.6 ± 26.2 (167)	− 8.4 ± 19.2 (161)	< 0.001
γGTP (IU/l)	43.0 [29.0, 69.0] (157)	33.0 [24.0, 56.0] (153)	− 6.0 [− 18.0, 0.0] (147)	< 0.001
Serum creatinine (mg/dl)	0.72 ± 0.19 (173)	0.75 ± 0.20 (168)	0.02 ± 0.09 (162)	0.001
Uric acid (mg/dl)	5.5 ± 1.5 (169)	5.1 ± 1.3 (174)	− 0.4 ± 0.8(162)	< 0.001
Amylase (IU/l)	62.2 ± 33.0 (116)	66.6 ± 33.8 (120)	3.3 ± 16.4 (104)	0.040
RBC (× 10^4^/μl)	487.0 ± 47.5 (160)	509.5 ± 45.6 (161)	23.0 ± 25.3 (151)	< 0.001
WBC (/μl)	7211.7 ± 1760.1 (160)	7174.3 ± 1856.8 (161)	27.5 ± 1369.5 (151)	0.81
Hemoglobin (g/dl)	14.5 ± 1.6 (160)	15.1 ± 1.6 (161)	0.6 ± 0.8 (151)	< 0.001
Hematocrit (%)	43.5 ± 4.2 (160)	45.8 ± 4.5 (161)	2.4 ± 2.6 (151)	< 0.001
Platelet (× 10^4^/μl)	24.3 ± 6.5 (160)	24.4 ± 6.4 (161)	0.1 ± 3.2 (151)	0.76
Urinary albumin/Cr (mg/g Cr)	14.9 [7.8, 32.4] (105)	16.0 [7.7, 34.5] (110)	0.3 [− 5.5, 7.0] (85)	0.64
Urinary albumin (mg/l)	16.6 [4.6, 47.6] (50)	13.3 [7.0, 39.6] (60)	− 2.2 [− 12.4, 0.7] (41)	0.07
Urinary creatinine (mg/dl)	129.0 ± 73.7 (48)	89.8 ± 47.3 (63)	− 33.2 ± 76.3 (42)	0.007
Systolic blood pressure (mmHg)	132.3 ± 14.1 (183)	129.8 ± 14.5 (181)	− 2.7 ± 13.7 (178)	0.011
Diastolic blood pressure (mmHg)	80.6 ± 11.9 (183)	79.0 ± 12.2 (181)	− 1.6 ± 10.4 (178)	0.047

Data are shown as mean ± SD (*n*) with *P* values by one-sample *t*-test, or median [Q1, Q3] (*n*) with *P* values by Wilcoxon signed-rank test

*HbA1c* glycosylated hemoglobin, *NGSP* national glycohemoglobin standardization program, BMI body mass index, *TC* total cholesterol, *HDL*-*C* high density lipoprotein cholesterol, *LDL*-*C* low density lipoprotein cholesterol, *TG* triglyceride, *AST* aspartate aminotransferase, *ALT* alanine aminotransferase, *γGTP* γ-glutamyl transpeptidase, *RBC* red blood cells, *WBC* white blood cells

### Adverse events

In the safety analysis set, a total of 18 adverse events were reported in 20 patients over the 14 weeks of the study and these are listed in Additional file 3. Adverse events included 1 case of frequent urination (0.5%), 1 case of bladder inflammation (0.5%), and 3 cases of vulvovaginal candidiasis (1.4%). No cases of hypoglycemia were noted.

## Discussion

In this study, we assessed the effects of dapagliflozin on treatment satisfaction for 14 weeks using the revised OHA-Q (OHA-Q ver. 2), a rating scale designed to assess satisfaction with OHA, including SGLT2 inhibitors. Because the original OHA-Q was developed before SGLT2 inhibitors became available, the original OHA-Q does not include questions regarding side effects specific to SGLT2 inhibitors. We added 3 new items (frequent urination, thirst, and discomfort with urination or genital pruritus), which are common side effects of SGLT2 inhibitors, to the original OHA-Q and developed the OHA-Q ver. 2. All of the 3 new items were included into the “somatic symptom” subscale. Internal consistency reliability was satisfactory for each subscale. As a result, we validated OHA-Q ver. 2 and the questionnaire was used for comprehensive evaluation of both positive and negative effects of SGLT2 inhibitors, and to compare OHAs, including SGLT2 inhibitors.

Dapagliflozin treatment was associated with treatment satisfaction, as shown by the significant increase in the OHA-Q ver. 2 total score in this study. We detected significant increases in 2 out of the 3 subscales (“satisfaction” and “somatic symptoms” subscales). The important results from our analysis of the questionnaire were in subscales 2 “somatic symptoms” and 3 “satisfaction”. The greatest variation in the scores was found just in the “satisfaction” subscale, showing the relationship with the loss of weight and decreasing HbA1c. The “satisfaction” subscale includes glycemic control, which may be reflected by the improvement in HbA1c levels as a significant correlation was detected between changes in HbA1c and the “satisfaction” subscale score. The “somatic symptoms” subscale consisted of 11 items, including the 3 newly added items. Positive significant changes were found in the following items: diarrhea, body weight, nausea, bodily swelling, and hypoglycemia. At 14 weeks there were no reports of these adverse events from patients and a significant reduction in body weight was observed. Regarding the 3 newly added items, we detected a significant reduction in frequent urination score; however, only 1 case of frequent urination as an adverse event was reported. We consider the number of reported adverse events was less than that actually felt by patients; this is evident based on the PRO scores. The remaining two items showed negative changes, implying a worsening of symptoms; however these changes were not significant. Previous observational study with SGLT2 inhibitor reported a higher prevalence of urinary tract infection particularly in female participants [30]; therefore, low urinary symptoms in the present study may be related to the higher percentage (65.4%) of the male participants. Those items were added to reflect the adverse events of SGLT2 inhibitors reported before. Despite the addition of the items reflecting negative side effects of SGLT2 inhibitors, the “somatic symptoms” subscale score showed a significant increase, indicating that dapagliflozin has an overall positive effect on the somatic symptoms of patients. The “treatment convenience” subscale did not show significant changes, indicating that treatment convenience did not worsen following the introduction of dapagliflozin therapy. These results show that the OHA-Q ver. 2 detected both negative and positive effects of dapagliflozin and indicate that, overall, dapagliflozin treatment improved treatment satisfaction among patients. Furthermore, reports indicate that treatment satisfaction correlate with treatment adherence and good adherence results in good glycemic control [31, 32]. In the present study, adherence with medication was 84.6% which is a relatively high rate [33, 34]. We expect that the introduction of dapagliflozin will improve compliance with drug therapy.

Consistent with previous reports [9, 35, 36], dapagliflozin treatment resulted in a significant decrease in HbA1c levels and body weight in this study. Body weight loss was associated with changes in the total score and the “satisfaction” subscale score in OHA-Q ver. 2. In a cross-sectional study, Nicole et al. reported that body weight loss increased satisfaction [28]. Although HbA1c improvement was not associated with change in the total score, it was associated with change in “satisfaction” subscale score of OHA-Q ver. 2. Aya et al. reported that there was no association between treatment satisfaction in the Diabetes Treatment Satisfaction Questionnaire (DTSQ) and HbA1c [37]. Changes in HbA1c levels may have a low impact on treatment satisfaction. Significant decreases were observed in abdominal circumference, body fat, systolic blood pressure, AST, ALT, and uric acid levels and a significant increase was observed in the hematocrit. Similar changes in these parameters have been previously reported [38–40].

Recently, several studies have evaluated treatment satisfaction or QOL of patients with T2DM treated with SGLT2 inhibitors [23–25, 27]. Grandy et al. reported that dapagliflozin treatment did not significantly improve QOL using EuroQoL-5 dimension (EQ-5D) [25]. Costel et al. showed that empagliflozin treatment significantly improved treatment satisfaction from baseline to 104 weeks using DTSQ states version [23]. No significant differences were found when comparing treatment with glimepiride [23] although the underlying reasons for these findings were not discussed by the authors. Grandy et al. and Traina et al. conducted weight-related QOL questionnaires, the Study to Help Improve Early evaluation and management of risk factors Leading to Diabetes Weight Questionnaire-9 (SHIELD-WQ-9) and the Impact of Weight on Quality of Life-Lite (IWQOL-Lite), respectively [24, 27]. In these studies, SGLT2 treatment significantly improved weight-related QOL. SHIELD-WQ-9 and IWQOL-Lite measure only the positive outcome of weight reduction. However, SGLT2 inhibitors have both positive and negative effects on patient QOL. Thus, these questionnaires are unfit for assessment of other side effects. Treatment satisfaction, QOL, and compliance with treatment, are affected by positive and negative effects of medication [23, 24, 27, 41, 42]. Therefore, it is important to assess both of positive and negative effects of SGLT2 inhibitors on treatment satisfaction and this has not been reported to date. OHA-Q ver. 2 can evaluate these effects, including weight changes and adverse events. OHA-Q ver. 2 is a specialized questionnaire designed to evaluate OHA treatment, including SGLT2 inhibitors. Therefore, it can evaluate treatment satisfaction among patients with T2DM treated with OHA more specifically than other questionnaires. This report presents new insights into treatment satisfaction among patients with T2DM, including those related to OHA side effects.

Our study has several limitations. First, this was single-arm study. Because there was no control arm, we could not exclude the effects of placebo. A subset of patients may reduce their body weight due to the effects of participating in the study and the body weight loss could decrease HbA1c, increasing treatment satisfaction in this study. Future studies need to evaluate satisfaction with dapagliflozin treatment in a randomized-controlled study. Second, the patients in this study used other OHAs, in addition to dapagliflozin; therefore, the results do not represent the effect of dapagliflozin alone but rather the combined effects of dapagliflozin and the other OHAs. However, the effects of other OHAs were considered to be limited because of the conditions for enrollment including no changes in the medication regime of participants for a minimum of 12 weeks prior to commencement of the study. Third, the study recruited Japanese patients, which may limit the generalizability of our findings to other ethnic groups.

## Conclusions

Dapagliflozin treatment was associated with improved treatment satisfaction among patients with T2DM, as measured using the OHA-Q ver. 2. Although dapagliflozin caused adverse events, including frequent urination, it improved the OHA-Q ver. 2 total score. Weight loss significantly improved treatment satisfaction. Conversely, changes in HbA1c levels did not result in a significant improvement in the total OHA-Q ver. 2 score. Because dapagliflozin reduced not only HbA1c but also body weight, it was useful for improving satisfaction.

## Additional files

**Additional file 1.** List of the 29 medical institutions participating in the study. The data on this file include the names of the 29 participating medical institutions in this multi-center study.

**Additional file 2.** Questions and choices in Oral Hypoglycemic Agent-Questionnaire (OHA-Q) ver.2. The data on this file consist of the questions and choices in the Oral Hypoglycemic Agent-Questionnaire (OHA-Q) ver.2.

**Additional file 3.** Adverse events. The data on this file consist of all adverse events.

## Abbreviations

ALT: alanine aminotransferase
AST: aspartate aminotransferase
BMI: body mass index
DPP-4: dipeptidyl peptidase-4
DTSQ: Diabetes Treatment Satisfaction Questionnaire
GLP-1: glucagon-like peptide-1
HbA1c: glycosylated hemoglobin
HRQOL: health-related quality of life
OHAs: oral hypoglycemic agents
OHA-Q: Oral Hypoglycemic Agent-Questionnaire
PRO: patient reported outcome
QOL: quality of life
SGLT2: sodium glucose cotransporters 2
T2DM: type 2 diabetes mellitus
