# Supplementary material for: Dapagliflozin improves treatment satisfaction in overweight patients with type 2 diabetes mellitus: a patient reported outcome study (PRO study)
Source: Diabetol Metab Syndr. 2018 Mar 1;10:11. doi: 10.1186/s13098-018-0313-x (PMC5831584; doi:10.1186/s13098-018-0313-x)
Supplement: Supplementary file 1 — Additional file 1. List of the 29 medical institutions participating in the study. The data on this file include the names of the 29 participating medical institutions in this multi-center study. [file 13098_2018_313_MOESM1_ESM.docx]

**Additional file 1**

**Title**:

Dapagliflozin improves treatment satisfaction in overweight patients with type 2 diabetes mellitus: a patient reported outcome study (PRO study)

**Short running title**:

Dapagliflozin effects on treatment satisfaction

**Authors**:

Hiroki Nakajima, Sadanori Okada, Takako Mohri, Eiichiro Kanda, Naoyuki Inaba, Yoko Hirasawa, Hiroaki Seino, Hisamoto Kuroda, Toru Hiyoshi, Tetsuji Niiya, Hitoshi Ishii

**Additional file 1. List of the 29 medical institutions participating in the study**

Shizuoka Saiseikai General Hospital, Plumeria DM Clinic, Seino Internal Medicine Clinic, Green Clinic, Matsuyama Shimin Hospital, Nishimura Memorial Hospital, Saitama Medical University Hospital, Japanese Red Cross Medical Center, Juntendo University Shizuoka Hospital, Kawai Clinic, Takagi Hospital, Nara Medical University, Matsubara Clinic, Fukuda Clinic, Osaka Kaisei Hospital, Juntendo University Hospital, Tokyo Rinkai Hospital, Yamamoto Clinic, Fuji-dori Owada Internal Clinic, Seiwa Clinic, Takahashi Clinic, Shinyoshida Clinic, Ikeda Clinic, Nakayama Clinic, Sangenjaya Hayakawa Clinic, Yamashita Internal Diabetes Medical clinic, Yokohama Rosai Hospital, Hamasaki Clinic, Kuroda Clinic.
