# Supplementary material for: Dapagliflozin improves treatment satisfaction in overweight patients with type 2 diabetes mellitus: a patient reported outcome study (PRO study)
Source: Diabetol Metab Syndr. 2018 Mar 1;10:11. doi: 10.1186/s13098-018-0313-x (PMC5831584; doi:10.1186/s13098-018-0313-x)
Supplement: Supplementary file 2 — Additional file 2. Questions and choices in Oral Hypoglycemic Agent-Questionnaire (OHA-Q) ver.2. The data on this file consist of the questions and choices in the Oral Hypoglycemic Agent-Questionnaire (OHA-Q) ver.2. [file 13098_2018_313_MOESM2_ESM.docx]

**Additional file 2**

**Title**:

Dapagliflozin improves treatment satisfaction in overweight patients with type 2 diabetes mellitus: a patient reported outcome study (PRO study)

**Short running title**:

Dapagliflozin effects on treatment satisfaction

**Authors**:

Hiroki Nakajima, Sadanori Okada, Takako Mohri, Eiichiro Kanda, Naoyuki Inaba, Yoko Hirasawa, Hiroaki Seino, Hisamoto Kuroda, Toru Hiyoshi, Tetsuji Niiya, Hitoshi Ishii

**Additional file 2. Questions and choices in Oral Hypoglycemic Agent-Questionnaire (OHA-Q) ver.2**

| 1. Do you ever forget to take your diabetes medication? (How many times a week?) | |
| --- | --- |
| 1. Never  3. Once or twice a week | 2. Almost never  4. At least three times a week |
| 2. Are you concerned about the size of the tablets, difficulty swallowing the tablets, etc., when taking diabetes medication? | |
| 1. Not concerned at all  3. Sometimes concerned | 2. Hardly concerned  4. Very concerned |
| 3. Is handling/carrying/preparing to taking diabetes medication troublesome? | |
| 1. Not troublesome at all  3. Sometimes troublesome | 2. Hardly troublesome  4. Very troublesome |
| 4. Are you concerned about being seen by others when taking diabetes medication outside of your home? | |
| 1. Not concerned at all  3. Sometimes concerned | 2. Hardly concerned  4. Very concerned |
| 5. Is it burden to eat meals at regular times in order to take diabetes medication? | |
| 1. Not a burden at all  3. Sometimes a burden | 2. Almost no burden  4. Very much of a burden |
| 6. Is being punctual in taking your diabetes medication and your meals troublesome? | |
| 1. Not troublesome at all  3. Sometimes troublesome | 2. Hardly troublesome  4. Very troublesome |
| 7. Is it burden to take diabetes medication at predetermined times? | |
| 1. Not a burden at all  3. Sometimes a burden | 2. Almost no burden  4. Very much of a burden |
| 8. Is the dosing frequency for diabetes medication a hassle? | |
| 1. Not a hassle at all  3. Sometimes a hassle | 2. Almost no hassle  4. Very much of a hassle |
| 9. Is it difficult to take diabetes medication outside of your home? | |
| 1. Not difficult at all  3. Sometimes difficult | 2. Hardly difficult  4. Very difficult |
| 10. Do you want to continue to take your current diabetes medication? | |
| 1. Yes, definitely  3. Not very much | 2. Yes  4. No, I would like to stop |
| 11. Are you concerned about passing gas or rumbling in your stomach? | |
| 1. Not concerned at all  3. Sometimes concerned | 2. Hardly concerned  4. Very concerned |
| 12. Are you concerned about diarrhea? | |
| 1. Not concerned at all  3. Sometimes concerned | 2. Hardly concerned  4. Very concerned |
| 13. Are you concerned about constipation? | |
| 1. Not concerned at all  3. Sometimes concerned | 2. Hardly concerned  4. Very concerned |
| 14. Are you concerned about weight gain? | |
| 1. Not concerned at all  3. Sometimes concerned | 2. Hardly concerned  4. Very concerned |
| 15. Are you concerned about readily becoming hungry? | |
| 1. Not concerned at all  3. Sometimes concerned | 2. Hardly concerned  4. Very concerned |
| 16. Are you concerned about having an upset stomach? | |
| 1. Not concerned at all  3. Sometimes concerned | 2. Hardly concerned  4. Very concerned |
| 17. Are you concerned about swelling of your body? | |
| 1. Not concerned at all  3. Sometimes concerned | 2. Hardly concerned  4. Very concerned |
| 18. Are you worried about hypoglycemia? | |
| 1. Not worried at all  3. Sometimes worried | 2. Hardly worried  4. Very worried |
| 19. Are you concerned about frequent urination? | |
| 1. Not concerned at all  3. Sometimes concerned | 2. Hardly concerned  4. Very concerned |
| 20. Are you concerned about thirsty? | |
| 1. Not concerned at all  3. Sometimes concerned | 2. Hardly concerned  4. Very concerned |
| 21. Are you concerned about discomfort with urination or genital pruritus? | |
| 1. Not concerned at all  3. Sometimes concerned | 2. Hardly concerned  4. Very concerned |
| 22. Are you satisfied with your current blood glucose control? | |
| 1. Very satisfied  3. Not very satisfied | 2. Generally satisfied  4. Dissatisfied |
| 23. Are you satisfied with your current treatment with the diabetes medication? | |
| 1. Very satisfied  3. Not very satisfied | 2. Generally satisfied  4. Dissatisfied |
|  | |
