# Supplementary material for: Dapagliflozin improves treatment satisfaction in overweight patients with type 2 diabetes mellitus: a patient reported outcome study (PRO study)
Source: Diabetol Metab Syndr. 2018 Mar 1;10:11. doi: 10.1186/s13098-018-0313-x (PMC5831584; doi:10.1186/s13098-018-0313-x)
Supplement: Supplementary file 3 — Additional file 3. Adverse events. The data on this file consist of all adverse events. [file 13098_2018_313_MOESM3_ESM.docx]

**Additional file 3**

**Title**:

Dapagliflozin improves treatment satisfaction in overweight patients with type 2 diabetes mellitus: a patient reported outcome study (PRO study)

**Short running title**:

Dapagliflozin effects on treatment satisfaction

**Authors**:

Hiroki Nakajima, Sadanori Okada, Takako Mohri, Eiichiro Kanda, Naoyuki Inaba, Yoko Hirasawa, Hiroaki Seino, Hisamoto Kuroda, Toru Hiyoshi, Tetsuji Niiya, Hitoshi Ishii

**Additional file 3. Adverse events**

| Adverse events | Frequency (%) |
| --- | --- |
| Any adverse events | 20 (9.3) |
| Vulvovaginal candidiasis | 3 (1.4) |
| Upper respiratory tract infection | 2 (0.9) |
| Myocardial ischemia | 2 (0.9)* |
| Positive type A influenza virus test | 1 (0.5) |
| Uncontrolled diabetes | 1 (0.5) |
| Miliaria | 1 (0.5) |
| Balanoposthitis | 1 (0.5) |
| Fatty liver | 1 (0.5) |
| Hemorrhagic diverticulitis | 1 (0.5)* |
| Sleep disorder | 1 (0.5) |
| Ureterolithiasis | 1 (0.5)* |
| Back pain | 1 (0.5) |
| Frequent urination | 1 (0.5) |
| Cellulitis | 1 (0.5) |
| Retinal detachment | 1 (0.5) |
| Depressed mood | 1 (0.5) |
| Bladder inflammation | 1 (0.5) |
| Hives | 1 (0.5) |
| Adverse events were analyzed in a safety analysis set (*n*=214). Frequencies are presented as number (percentage) of patients for each adverse event. Asterisks show serious adverse events. | |
